# T Cell Repertoire Abnormality in Immunodeficiency Patients with DNA Repair and Methylation Defects

**DOI:** 10.1007/s10875-021-01178-1

**Published:** 2021-11-25

**Authors:** Mingyan Fang, Zheng Su, Hassan Abolhassani, Wei Zhang, Chongyi Jiang, Bochen Cheng, Lihua Luo, Jinghua Wu, Shiyu Wang, Liya Lin, Xie Wang, Longlong Wang, Asghar Aghamohammadi, Tao Li, Xiuqing Zhang, Lennart Hammarström, Xiao Liu

**Affiliations:** 1 BGI-Shenzhen, Shenzhen, 518083 China; 2 Division of Clinical Immunology at the Department of Laboratory Medicine, Karolinska Institutet at Karolinska University Hospital Huddinge, 141 86 Stockholm, Sweden; 3 School of Biotechnology and Biomolecular Sciences, Faculty of Science, The University of New South Wales, Sydney, NSW Australia; 4 Research Center for Immunodeficiencies, Pediatrics Center of Excellence, Children’s Medical Center, Tehran University of Medical Sciences, Tehran, Iran; 5 Department of Biosciences and Nutrition, Karolinska Institutet, Stockholm, Sweden; 6 Department of Computer Science, City University of Hong Kong, Hong Kong, 999077 China; 7 Tsinghua Shenzhen International Graduate School, Tsinghua University, Shenzhen, 518055 China

**Keywords:** Inborn errors of immunity, Primary immunodeficiency diseases, TCR repertoire analysis, Immunogenetics, Ataxia telangiectasia mutated, DNA repair, DNA methylation

## Abstract

Both DNA damage response and methylation play a crucial role in antigen receptor recombination by creating a diverse repertoire in developing lymphocytes, but how their defects relate to T cell repertoire and phenotypic heterogeneity of immunodeficiency remains obscure. We studied the TCR repertoire in patients with the mutation in different genes (*ATM, DNMT3B*, *ZBTB24*, *RAG1*, *DCLRE1C*, and *JAK3*) and uncovered distinct characteristics of repertoire diversity. We propose that early aberrancies in thymus T cell development predispose to the heterogeneous phenotypes of the immunodeficiency spectrum. Shorter CDR3 lengths in ATM-deficient patients, resulting from a decreased number of nucleotide insertions during VDJ recombination in the pre-selected TCR repertoire, as well as the increment of CDR3 tyrosine residues, lead to the enrichment of pathology-associated TCRs, which may contribute to the phenotypes of ATM deficiency. Furthermore, patients with *DNMT3B* and *ZBTB24* mutations who exhibit discrepant phenotypes present longer CDR3 lengths and reduced number of known pathology-associated TCRs.

**Supplementary Information:**

The online version contains supplementary material available at 10.1007/s10875-021-01178-1.

## Introduction

Inborn errors of immunity (IEI) or primary immunodeficiency diseases (PIDs) constitute a group of highly heterogeneous genetic disorders caused by defects in the immune system, predisposing individuals to an increased frequency and severity of infections, immune dysregulation, autoimmune manifestations, and malignancies [1]. The establishment of adaptive immunity in a selected group of IEI patients with DNA repair and methylation defects might be perturbed, resulting in a restricted diversity of the immune repertoire, and increased susceptibility to infections [2].

The diversity of antigen receptor repertoires is generated by recombination of the variable (V), diversity (D), and joining (J) gene segments. And the diversity is further augmented by junctional diversity (non-templated (N) nucleotide additions in the V-D and D-J junctions inserted by terminal deoxynucleotidyl transferase (TdT) and random deletion of nucleotides at the recombining edges as a consequence of asymmetric hairpin opening by ARTEMIS) [3–5]. The productive T cell receptor (TCR) repertoire further undergoes a selection process in the thymus and subsequently through interaction with antigens. During V(D)J recombination, DNA double-strand breaks (DSBs) near the V, D, and J coding gene segments and recombination signal sequences [6] are introduced by the recombination activating gene (*RAG1* and *RAG2*) complex, and mutations in these two genes can lead to severe combined immunodeficiency (SCID) [7].

In addition, epigenetic modifications play a vital role in changing the recombination signal sequences’ accessibility to the recombinase [8], as well as recruitment and stable binding of the recombination complex [9]. The epigenetic plasticity, involved in lymphocyte development and activation, plays a role in the pathogenesis of immune diseases [9]. For instance, immunodeficiency, centromere instability, and facial anomalies syndrome (ICF) is a rare autosomal recessive immunodeficiency, which is characterized by recurrent and often fatal respiratory infections and juxtacentromeric heterochromatin instability. The main identified pathogenic defects in ICF syndrome are mutations in the DNA methyltransferase 3B (*DNMT3B*) gene and the zinc finger and BTB domain containing 24 (*ZBTB24*) gene. DNMT3B acts as a de novo DNA methyltransferase, initially acting during early development in CpG dense regions [10]; mutations result in severe chromosomal rearrangements in lymphocytes [11]. *ZBTB24* encodes a member of a family of transcription factors, which have regulatory roles in B and T cell differentiation [12].

DSBs are repaired by two major pathways, which are homologous recombination (HR) and non-homologous DNA end joining (NHEJ). The fastest repair mechanism of pathologic and physiologic DSBs in humans is NHEJ, the only DNA repair mechanism acting during V(D)J recombination. Patients lacking normal NHEJ are sensitive to DNA damage and prone to develop severe immunodeficiency, often associated with growth retardation and neurological abnormalities [13, 14]. Ataxia-telangiectasia mutated kinase (ATM) was found to play a role in both NHEJ and HR pathways [15–17]; it is a member of the phosphatidylinositol 3 kinase (PI3Ks) family and can modify hundreds of proteins at specific sites during the DNA damage response (DDR) [18]. ATM can be activated by the MRN (MRE11–RAD50–NBS1) complex [19], and the activated ATM induces chromatin changes around the DSB sites and promotes the recruitment of checkpoint and repair factors. ATM directly or indirectly (by phosphorylation of the MRN complex) spreads the damage signal and promotes DNA repair, cell cycle arrest, and apoptosis when it is recruited to the DSBs [19–21]. *ATM* is the causing gene of ataxia-telangiectasia (AT) (OMIM #208900), which is an autosomal recessive inherited disease, and it is associated with recurrent upper and lower respiratory tract infections and a trend for increased susceptibility to cancers.

Analyzing antigen receptor repertoires with high-throughput sequencing can characterize the clonality and diversity of repertoire. Immune repertoires as a distinctive feature of antigen receptor (V(D)J) rearrangements have been explored in several IEIs [22], including RAG deficiency (TCRβ and BCR) [23], Ataxia-telangiectasia (AT) (TCRα and BCR) [24, 25], Cernunnos deficiency (TCRβ, TCRδ, and BCR) [4], Wiskott-Aldrich syndrome (TCRβ) [26], and common variable immunodeficiency (CVID) (TCRβ, BCR) [27, 28].

However, the potential role of *DNMT3B* and *ZBTB24* in the regulation of T cell development remains undefined, and the TCRβ repertoire in AT patients has not been explored yet. Furthermore, little is known about how the defect of these genes contributes to the diverse phenotypes of immunodeficiency. We, therefore, performed deep sequencing of the TCRβ complementarity-determining region 3 (CDR3) regions on a selected group of IEI patients with DNA repair and methylation defects, which are patients mutated in *ATM*, *DNMT3B*, *ZBTB24*, *RAG1*, *DCLRE1C*, and *JAK3*, to investigate the characteristics of their repertoire.

The characteristics of the TCR repertoire observed in our study provide novel insights into the role of different components of the DNA repair and methylation machinery in TCR repertoire abnormalities. We identified an additional role for *ATM* in the contribution to junctional diversity during V(D)J recombination, resulting in a shorter CDR3 length which may contribute to the generation of pathology (pathogen, cancer, and autoimmunity)-associated T cell receptors. Furthermore, for the first time, we investigated the potential function of the poorly characterized *ZBTB24* and *DNMT3B* genes in shaping the T cell repertoire, thus shedding light on the observed clinical heterogeneity.

## Materials and Methods

### Human Subjects

Genomic DNA from 19 IEI patients with evidence of DNA repair defects and radiosensitivity and 14 age-matched healthy donors was extracted from peripheral blood, and 18 patients underwent whole-exome sequencing (WES). All patients and normal individuals were used for TCRβ repertoire sequencing. Approvals were obtained from the human research ethics committees at the Institutional Review Boards of the Karolinska Institutet, the Tehran University of Medical Sciences, and the Institute of Research in Biomedicine of BGI-Shenzhen. Informed consent for the performed tests was obtained from all patients and/or their parents. All patients were diagnosed as IEI based on the updated clinical diagnostic criteria of the European Society for Immunodeficiencies (ESID) [29, 30].

### Whole-Exome Sequencing

Agilent SureSelect Human All Exon V5 (50 Mb, Agilent Technologies, Santa Clara, CA) were used for whole-exome capture and Illumina HiSeq2000, and 90 bases paired-end sequencing (Illumina) was performed for exon library sequencing. The candidate gene screening pipeline of WES data was performed as previously described [31]. Burrows-Wheeler Aligner (BWA), Genome Analysis Toolkit (GATK), and Variant Effect Predictor (VEP) were carried out for reads alignment to the GRCh37/hg19 human reference genome, variants calling, and annotation, respectively. Public databases including the 1000 Genomes Project (KG), Exome Variant Server (ESP), Exome Aggregation Consortium (ExAC), and The Genome Aggregation Database (gnomAD) were used to identify and remove polymorphisms. Copy number variants (CNV) were detected by ExomeDepth.

### Sequencing of TCRβ Repertoires

Multiplex primers and two complete sequencing adapter primers described before [3, 32] were used to amplify the CDR3 sequences of TCRβ genes for equal amounts of DNA samples (1.2 µg) from all TCR subjects. AMPure XP (Beckman, A63882) was used to clean the first 15 cycles amplicons and purified PCR products were then amplified for a second round (25 cycles) with a pair of communal primers. Libraries were loaded onto a BGIseq500 (BGI-Shenzhen, Shenzhen, China) and underwent 200 bp single end to perform sequencing. A mean of 5,971,100 (range 1,345,371–20,526,738) total reads and 5,159,138 (range 824,569–17,542,667) clean reads were obtained for the sequenced samples.

### Bioinformatics Analyses of TCRβ Repertoires

TCR sequencing data were analyzed using iMonitor [3]. The final alignment result was obtained according to the optimal score after two rounds of Basic Local Alignment Search Tool (BLAST) [3], while the International ImMunoGenneTics database (IMGT, www.imgt.org) was used as a reference. In-frame and out-of-frame determinations; V, D, and J segments usage calculation; deletion/insertion nucleotide; and amino acid sequence determination at the rearrangement were performed with default parameters as described previously [3]. The rearrangement frame was labeled as “out-of-frame” if the sequence contains a stop codon or has a length of non-multiple of three (frameshift); otherwise, it was labeled as “in-frame”. Evenness is best expressed as a partial order, and this post-structure can adequately be illustrated by Shannon–Wiener’s diversity (or entropy) index (H’) and the Gini evenness coefficient (G’) measured by considering three basic requirements: permutation invariance, scale invariance, and the transfer principle.

### Analysis of Pathology-Associated CDR3 Sequence

In total 20,814 pathology-associated CDR3 records of humans (Table S1) were collected from a manually curated database of T and B cell receptors targeting known antigens (TBAdb) (https://db.cngb.org/pird/tbadb/) and online databases McPAS-TCR (http://friedmanlab.weizmann.ac.il/McPAS-TCR/), where all records were derived from a manual literature search. We used all CDR3β sequences as a reference to perform alignment for all studied patients, and identical CDR3 sequences that are absolutely aligned to the reference were then calculated and annotated as specific for various pathogens.

### Statistical Analysis

Statistical analysis and data visualization was performed using R (R version 3.3.2). To account for the different sequencing depths among samples, normalization by a total number of reads was performed. Metrics calculated from normalized one million reads of data were used to describe the CDR3 sequences of each sample, including Shannon’s H index for measurement of repertoire diversity, Gini coefficiency of clonality, frequency of top 100 CDR3 clones, Pielou’s evenness index of V-J pairing, frequency of in-frame and out-of-frame rearrangements, nucleotide composition of the CDR3 sequences, and V gene usage. Differences in these metrics between groups were investigated, and their statistical significance was tested by two-sided Wilcoxon Rank Sum Test. In the V gene usage comparison, Bonferroni correction was used for multiple tests correction. Length distribution of the CDR3 sequences and insertion/deletion sequences were further examined to investigate the patterns in each group during pre-selection and post-selection of TCRβ rearrangement, where two-sided bootstrap Kolmogorov–Smirnov test was used for testing the distribution differences and one-sided Wilcoxon Rank Sum Test was used to test for the significance of length difference between each group. PCA analysis and visualization were performed using the built-in R function and function from additional packages ggplot2 downloaded from CRAN (https://cran.r-project.org/). Adjustments of all statistical tests have been made to multiple testing corrections; *p* values ≤ 0.05 or FDRs ≤ 0.1 were taken to be significant.

## Results

### Patient’s Clinical Characteristics and Molecular Diagnosis

After performing WES, 19 IEI patients that were found to have mutations in DNA repair and methylation genes were recruited into this study. The pathogenicity of all disease attributable gene variants was re-evaluated using the updated guideline for interpretation of molecular sequencing by the American College of Medical Genetics and Genomics (ACMG) criteria [33, 34], considering the allele frequency in the population database, computational data, immunological data, and clinical phenotyping. The causative genetic variations later were confirmed by Sanger sequencing and segregation validation in each affected pedigree (Table 1). The patients consisted of 13 cases with a clinical diagnosis of atypical combined immunodeficiency (CID) and 6 patients with AT [35]. According to the molecular diagnosis, we subdivided our patients into four groups including atypical T^+^ SCID (*n* = 3), AT (*n* = 6), ICF1 (*n* = 6), and ICF2 (*n* = 4). We classified patients with a hypomorphic mutation in NHEJ components (*RAG1* hypomorphic with 48% residual activity [36], *DCLRE1C-* OMIM #602450) and a patient with mutation in *JAK3* (OMIM #600802) [37] as atypical SCID, due to incompatibility with the standard diagnostic criteria of the European Society for Immunodeficiencies (ESID) and recent phenotypic classification of the International Union of Immunological Societies (IUIS) [38]. The median age at the time of the study was not significantly different between the four groups (9.2, 10.6, 9.1, and 13.2 years, respectively).

**Table 1:** Causative genes of studied subjects

ID	Group		Variation type	Consequence	HGNC (symbol)		HGVSc	HGVSp	Exon (intron)	Zygosity
P1		Atypical SCID	snv	missense_variant	*RAG1*	c.1073G > A		p.Cys358Tyr	'2/2'	Hom
P2	Atypical SCID		snv	missense_variant	*DCLRE1C*	c.386G > T		p.Gly129Val	'6/14'	Hom
P3	Atypical SCID		snv	missense_variant	*JAK3*	c.2164G > A		p.Val722Ile	'16/24'	Hom
P4	AT		InDel	frameshift_variant	*ATM*	c.6259delG		p.Glu2087LysfsTer9	'43/63'	Comp Het
			snv	stop_gained	*ATM*	c.6658C > T		p.Gln2220Ter	'46/63'	
P5	AT		InDel	frameshift_variant	*ATM*	c.6629delA		p.Gln2210ArgfsTer25	'46/63'	Hom
P6	AT		snv	missense_variant	*ATM*	c.6047A > G		p.Asp2016Gly	'41/63'	Hom
P7	AT		snv	missense_variant	*ATM*	c.6047A > G		p.Asp2016Gly	'41/63'	Comp Het
			snv	splice_donor_variant	*ATM*	c.6198 + 1G > A			'42/62'	
P8	AT		snv	stop_gained	*ATM*	c.3102 T > G		p.Tyr1034Ter	'21/63'	Hom
P9	AT		InDel	large deletion	*ATM*				1 ~ 2/63	Hom
P10	ICF1		snv	missense_variant	*DNMT3B*	c.2166 T > A		p.Asp722Glu	'20/23'	Hom
P11	ICF1		snv	missense_variant	*DNMT3B*	c.2166 T > A		p.Asp722Glu	'20/23'	Hom
P12	ICF1		snv	missense_variant	*DNMT3B*	c.2166 T > A		p.Asp722Glu	'20/23'	Hom
P13	ICF1		snv	missense_variant	*DNMT3B*	c.2428G > T		p.Gly810Cys	'23/23'	Hom
P14	ICF1		snv	missense_variant	*DNMT3B*	c.2166 T > A		p.Asp722Glu	'20/23'	Hom
P15	ICF1		snv	missense_variant	*DNMT3B*	c.1871A > G		p.Tyr624Cys	'17/23'	Hom
P16	ICF2		InDel	frameshift_variant	*ZBTB24*	c.795dupA		p.Asp266ArgfsTer28	'2/7'	Hom
P17	ICF2		snv	missense_variant	*ZBTB24*	c.1148G > C		p.Cys383Ser	'4/7'	Hom
P18	ICF2		snv	missense_variant	*ZBTB24*	c.1148G > C		p.Cys383Ser	'4/7'	Hom
P19	ICF2		snv	missense_variant	*ZBTB24*	c.1224C > G		p.Cys408Trp	'5/7'	Hom

*snv* single nucleotide variants, *InDel* insertions and deletions, *HGNC* Human Gene Nomenclature Committee, *HGVSc* Human Genome Variation Society codon, *HGVSp* Human Genome Variation Society polypeptide, *Hom* homozygous, *Comp Het* compound heterozygous

The clinical and immunological phenotypes of the patients (8 male, 11 female) are described in Table 2 and Fig. S1, where the AT patients presented a more severe phenotype than the other patients, even as compared to the atypical SCID group. In our AT patients, low counts of lymphocytes, absolute T and B cell numbers, and total CD4^+^ and CD8^+^ T cell numbers were observed, with a mean value of 1,663 lymphocytes, 1,295 T cells, 135 B cells, 723 CD4^+^ cells, and 512 CD8^+^ cells per microliter (µL), respectively (Table 2 and Fig. S1A-E), which is consistent with previous studies [25, 39]. The lymphocyte counts in AT patients was significantly reduced as compared with the other groups (*p* = 0.0238, Lymphocyte _AT_ vs Lymphocyte _Atypical SCID_, *p* = 0.0043, Lymphocyte _AT_ vs Lymphocyte _ICF1_ and 0.0260 CD8^+^ cells _AT_ vs CD8^+^ cell _ICF1_) (Table 2 and Fig. S1A,and E). Correlation analysis shows that the T cell count, as expected, is positively correlated with the concentration of lymphocytes, CD4^+^ and CD8^+^ cells in all patients (Fig. S1G-I).

**Table 2:** Demographic data and clinical characteristics of studied subjects with DNA repair defects

ID	Clinical diagnosis	Age of onset	Infections	Autoimmunity	Other complication	Lymphocytes (/µL)	T cells (/µL)	CD4 + cells (/µL)	CD8 + cells (/uL)	(% of CD4 + cells)	B cells cells (/µL)
P1	Atypical CID	4y	URI, LRI	AIN	LAP, HSM, GR, ED	4,000	1,501	825	310	55.2	109
P2	Atypical CID	15y	URI, LRI	ITP	ENT	3,600	2,120	612	1,510	17.2	72
P3	Atypical CID	1.5y	URI, LRI, CNS	-	LAP, ED	2,464	1,374	616	665	25.1	714
P4	AT	1.5y	URI	-	Dystonia	2,250	1,720	1,030	680	45.7	230
P5	AT	2.5y	URI, LRI	-	Asthma	1,740	1,449	748	609	42.9	152
P6	AT	2y	URI, LRI	-	-	1,872	1,530	820	672	43.8	135
P7	AT	1y	URI, LRI	-	-	1,680	1,390	713	550	51.2	107
P8	AT	2y	URI, LRI	-	Paralysis	1,330	950	520	375	39.0	110
P9	AT	0.5y	URI, LRI	-	HSM, ED	1,108	731	509	188	46.2	75
P10	Atypical CID	1.5y	URI, LRI	-	Skin lesions, ENT	3,350	2,950	2,057	836	61.4	114
P11	Atypical CID	7y	URI, LRI	-	LAP	4,200	3,360	1,512	1,764	36.0	418
P12	Atypical CID	4y	URI, LRI	-	-	5,400	4,025	2,590	1,677	47.9	810
P13	Atypical CID	1y	URI, LRI	JRA	PDA	1,915	1,263	574	708	30.0	445
P14	Atypical CID	10y	URI	-	Skin lesions, ENT	2,280	1,478	785	696	34.4	547
P15	Atypical CID	0.5y	URI, LRI	-	-	2,491	1,395	822	498	32.9	810
P16	Atypical CID	5y	URI	-	ENT, seizure	3,700	2,845	1,950	854	52.7	726
P17	Atypical CID	22y	URI	-	Nystagmus	1,557	1,022	705	309	68.9	250
P18	Atypical CID	8y	URI, LRI	GH, ITP, JRA	HSM	1,932	1,375	347	985	17.9	385
P19	Atypical CID	1.5y	URI	-	ENT	2,950	2,440	1180	1,093	40	74

*URI* upper respiratory tract infections, *LRI* lower respiratory tract infections, *AIN* autoimmune neutropenia, *LAP* lymphadenopathy, *HSM* hepatosplenomegaly, *GR* granulomatous lesion, *CID* combined immunodeficiency, *ITP* idiopathic thrombocytopenic purpura, *ENT* enteropathy, *CNS* central nervous system infection, *AT* ataxia telangiectasia, *ED* early death, *PDA* persistent ductus arteriosus, *GH* growth hormone deficiency, *JRA* juvenile rheumatoid arthritis

### Restriction of TCR Repertoire Correlates with the Pathogenic Cause and the Clinical Phenotype

It is generally thought that TCR repertoires are dynamically changed and that diversity declines with aging [40–42] and thus, age-matched controls have usually been used in immune repertoire analyses [39, 43]. To investigate the V(D)J recombination defect and analyze the TCRβ repertoire in patients with different pathogenic backgrounds, we compared their repertoire diversity and complexity using next-generation sequencing in four groups of patients together with 14 age-matched controls.

TCRβ rearrangements were detected in all patients. To evaluate the diversity and clonality of repertoire, we introduced some economic indexes, such as Shannon’s H index [3] and Gini coefficient, to measure repertoire diversity (diversity of CDR3, the V/J gene usage, and V-J pairing) and to evaluate repertoire homogeneity, respectively. Of note, immune repertoires in patients with atypical SCID showed a restricted diversity reflecting the hypomorphic nature of the identified variants in our patients reported previously [35].

**Fig. 1:**
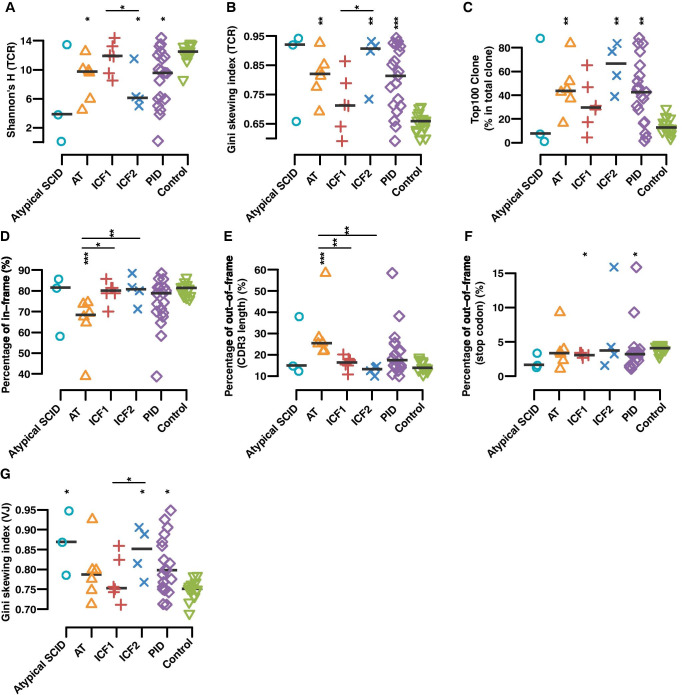
TCRβ repertoire diversity correlates with the severity of the clinical phenotype. **A**, **B** Scatter plot of Shannon’s H index of diversity (considering both the number of total sequences and clonal size distribution in the overall repertoire) (**A**) and Gini skewing index of unevenness (direct measure of TCR distribution among T cells to track subtle changes of the TCR repertoire among distinct populations of T cells) (**B)** were calculated to represent the quantification of the diversity and unevenness of TCR in different groups, where IEI represents all four patient groups taken together. **C** Percentage of top 100 abundant clones among the total TRB clones in patient groups and health individuals. **D**, **E**, **F** Percentage of in-frame CDR3 (**D**) and out-of-frame CDR3 rearrangement that either divided into those containing a stop codon within their sequence (**E**) or sequences with length of non-multiple of three (**F**). **G** Gini’s skewing index of V-J paring. The asterisk above each group indicate the significance tests between each group and normal controls, and asterisk above the line are *p*-values between two groups in the ends of line (*p* ≤ 0.05 *, *p* ≤ 0.01 **, *p* ≤ 0.001 ***, *p* ≤ 0.0001 ****, two-sided Wilcoxon Rank Sum Test followed by multiple testing correction)

The TCRβ repertoire of the AT patients was highly restricted, not just concerning the repertoire diversity as shown by a low Shannon’s H index (Fig. 1A), but also for the evenness of clonality, represented by a higher Gini coefficient (Fig. 1B) when compared to healthy controls. The total frequency of the top 100 CDR3 clonotypes (Fig. 1C) was significantly higher in the AT patients, and a reduction of the total number of unique clones (*p* = 0.016) was also observed in the AT group as compared to controls (Table S2). A lower ratio of in-frame CDR3 rearrangement (Fig. 1D) and a high proportion of out-of-frame rearrangements due to frameshifts (Fig. 1E) in AT patients versus healthy controls and the other DNA repair and methylation deficient patient groups were also observed.

In contrast, no significant change of the TCRβ repertoire was observed when the ICF1 group was compared to controls, except that ICF1 patients showed a significantly lower percentage of out-of-frame TCR genes containing a stop codon within their CDR3 (*p* = 0.036 versus controls, Fig. 1F). Interestingly, when comparing the ICF2 to the ICF1 group (Wilcoxon Rank Sum Test, Fig. 1A-B, and G), we observed significant differences in clone diversity (Shannon’s H, unevenness (Gini skewing index of TCR), and Gini skewing index of V-J pairing). This phenomenon correlates with the reduced number of lymphocytes in the peripheral blood of the ICF2 patients (Table 1 and Fig. S1A and B). Most notably, the TCRβ repertoire of the ICF2 group was characterized by restriction of diversity (Fig. 1A–B) when compared with normal individuals or ICF1 patients, representing a higher percentage of the top 100 clonotypes (Fig. 1C) and a smaller number of unique clones (Table S2) in comparison with healthy controls.

### Skewed V Gene Usage and V-J Pairing

TCRβ repertoire diversity and clonality are initially created by random V(D)J recombination, further enhanced by Insertion/Deletion (InDel) of nucleotides in the junctional regions between the V, D, and J segments and followed by selection according to the receptor fitness and response to self-antigens [44]. Previously, twin studies have suggested that V gene segment usage in TCRβ is predominantly affected by the germ-line locus factor of recombination machinery [45] and preferred usage of specific V genes that might influence clonotypic expansion in response to antigens.

To determine the role of different underlying pathogenic variants in individual V gene usage or V-J pairing, we first compared the spectrum of V gene usage in unique TCRβ clones. Marked reduction of *TRBV19* usage in the AT group (*p* < 0.001, Fig. S2A) and *TRBV7-3* usage in the ICF2 group (*p* < 0.001, Fig. S2B) in comparison to normal individuals was observed.

A considerable decrease of the repertoire diversity was also demonstrated in ICF2 patients when compared to normal individuals or ICF1 patients and to a lesser extent in the AT patients using Gini’s skewing index of V-J paring (Fig. 1G) and treemaps, a graphical representation of the diversity of V-J pairing (Fig. S3). The low diversity of V-J pairing was also observed in the atypical SCID patient group which might be related to the pathogenicity of the identified variants in NHEJ repair function presenting a late-onset atypical disease, despite normal lymphocyte subsets.

### Aberrant CDR3 Length

**Fig. 2:**
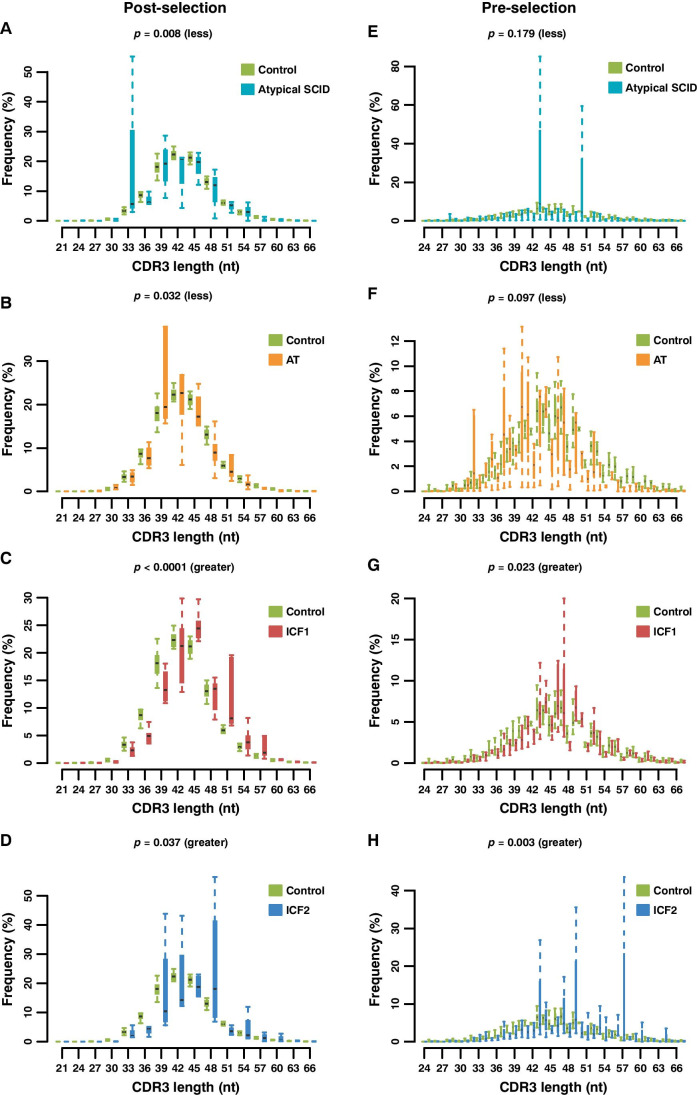
Distribution of the length of unique post-selection (in-frame, in nucleotides) and pre-selection CDR3 sequences (out-of-frame, in nucleotides). Distributions of in-frame CDR3 length show a bias toward shorter lengths in atypical SCID (**A**) and AT (**B**) patients and longer lengths in ICF1 (**C**) and ICF2 (**D**) compared to all normal samples. Similar results are observed in out-of-frame CDR3 length which present a bias toward either shorter lengths in atypical SCID (**E**) and AT (**F**) patients or longer lengths in ICF1 (**G**) and ICF2 (**H**) compared to healthy donors. One-sided Wilcoxon Rank Sum Test for the significance of length difference between each group pair

CDR3 length and composition usually play a crucial role in adaptive immune responses to a variety of non-self-antigens as well as an increased risk of recognizing self-antigens in autoimmune diseases [46, 47]. We analyzed the length of the productive CDR3 (in-frame rearrangements) in different IEI groups and the differences in distribution compared to controls. Substantial reduction of the CDR3 length was observed in both AT and atypical SCID patients versus controls. By contrast, the CDR3 length distribution was increased both in the ICF1 and ICF2 patients compared with the healthy individuals (Fig. 2A–D and Fig. S4A).

It is generally thought that TCR repertoire bias can be introduced during the VDJ gene recombination process in the thymus, which could be evaluated by the non-productive rearrangements (out-of-frame CDR3s), or after the positive/negative selection in the thymus and antigenic selection in the periphery [43]. Furthermore, pre-selection of TCR formation (out-of-frame rearrangements) together with T cell selection in the thymus both depend on genetic factors. In order to better understand whether the aberrant CDR3 length happens before or after the selection, we repeated the CDR3 length distribution analyses for out-of-frame rearrangements, and a very similar trend was observed (Fig. 2E–H and Fig. S4B). It, therefore, implies that the pre-selection rearrangement gave rise to the aberrant CDR3 length.

The aberrant CDR3 length profile from patients could be the result of deviations of an InDel (either N-nucleotides inserted by terminal deoxynucleotidyl transferase (TdT) or deletions at the ends of V, D, and J segments). Therefore, to further examine the role of DNA repair defects genes in junctional diversity, we analyzed the variation of deletions or insertions in cases and controls, by calculating the length distribution of 6 segments (3’V, 5’D, 3’D, 5’J deletions and V-D, D-J insertions) in out-of-frame and in-frame rearrangements, separately.

**Fig. 3:**
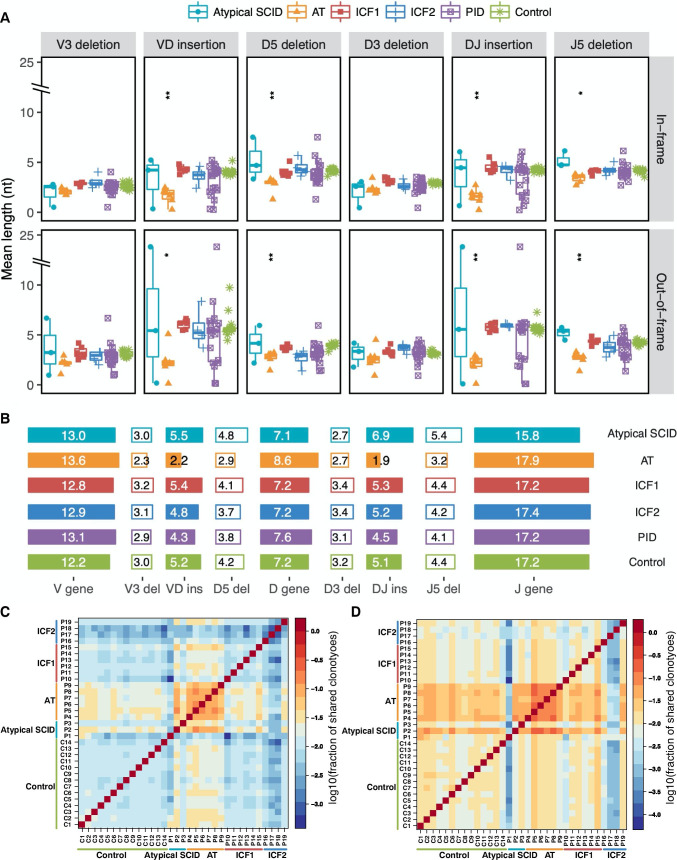
Altered InDel length distribution in DNA repair/methylation defect patient groups. **A** The length of 6 segments of 3’V, 5’D, 3’D and 5’J deletion, V-D and D-J insertion sequences of AT patients are shorter than healthy controls, both in out-of-frame or in-frame rearrangements. The reduction of insertion length is more remarkable than the length of deletion decrease. **B** Schematic diagram shows the average length of V, D, and J gene and InDel in different patient groups. **C**, **D** The heatmap represents fraction of shared unique (**C**) and total (**D**) CDR3 sequences within sample pairs, where the denominator is the average CDR3 sequence count of two samples. The asterisk above each group indicates the significance level of the differences between each group and normal controls (*p* ≤ 0.05 *, *p* ≤ 0.01 **, *p* ≤ 0.001 ***, *p* ≤ 0.0001 ****, two-sided Wilcoxon Rank Sum Test followed by false discovery rate control)

Shorter deletions at the end of the V, D, and J segments result in an increased V, D, and J gene length in AT patients (Fig. S4, C-E, Table S3, 4). At the same time, in these patients, fewer nucleotides (N region) added to the sites of V-D and D-J junctions were also observed in both out-of-frame (Fig. S5 and Fig. 3A) and in-frame (Fig. S6 and Fig. 3A) rearrangements, and the same observation precedent was reported in XLF deficiency patients [4]. However, a reduced insertion length in the AT patients plays a more significant role than reduction in deletion length and leads to a shorter mean CDR3 length (Fig. 3A–B). The average insertion size is usually around 2–5 bp per coding joint which is optimal for base pair recognition patterns [48, 49]. Here, we found a statistically significant decrease in AT patients for both out-of-frame (average 3.63nt decreased) and in-frame (around 2.55nt reduced) versus controls (Fig. 3A–B and Table S4).

The InDel length distribution was significantly different between ICF patients (ICF1 and ICF2) and healthy controls (Table S3). A longer V gene length in CDR3 region (Fig. S4C) contributes to the increased mean CDR3 length in both the ICF1 and ICF2 group (Fig. S4A-B ).

### Decreased CDR3 Length and Insertion Size Increase the Shared Clonotypes

To characterize the level of sequence similarity of the TCRβ repertoires within and between different diseases, we also calculated the fraction of shared identical clonotypes between sample pairs. The calculation revealed that more unique and total clonotypes were shared within the AT group, and an opposite trend was observed in ICF syndrome patients (Fig. 3C–D). The observation is consistent with previous reports that the shared clonotypes are characterized by a small size of insertion and the subsequent shorter CDR3 length, which increases the likelihood of two TCRβ sequences being identical [45, 50, 51]. Although AT patients and controls also have a high fraction of shared identical clones, the ratio of shared clonotypes in controls was smaller than what was found between AT patients (Fig. 3D), which might be due to a limited number of unique clones existing in the AT patients compared to controls (Table S2).

### Abnormality of CDR3 Amino Acids Composition

**Fig. 4:**
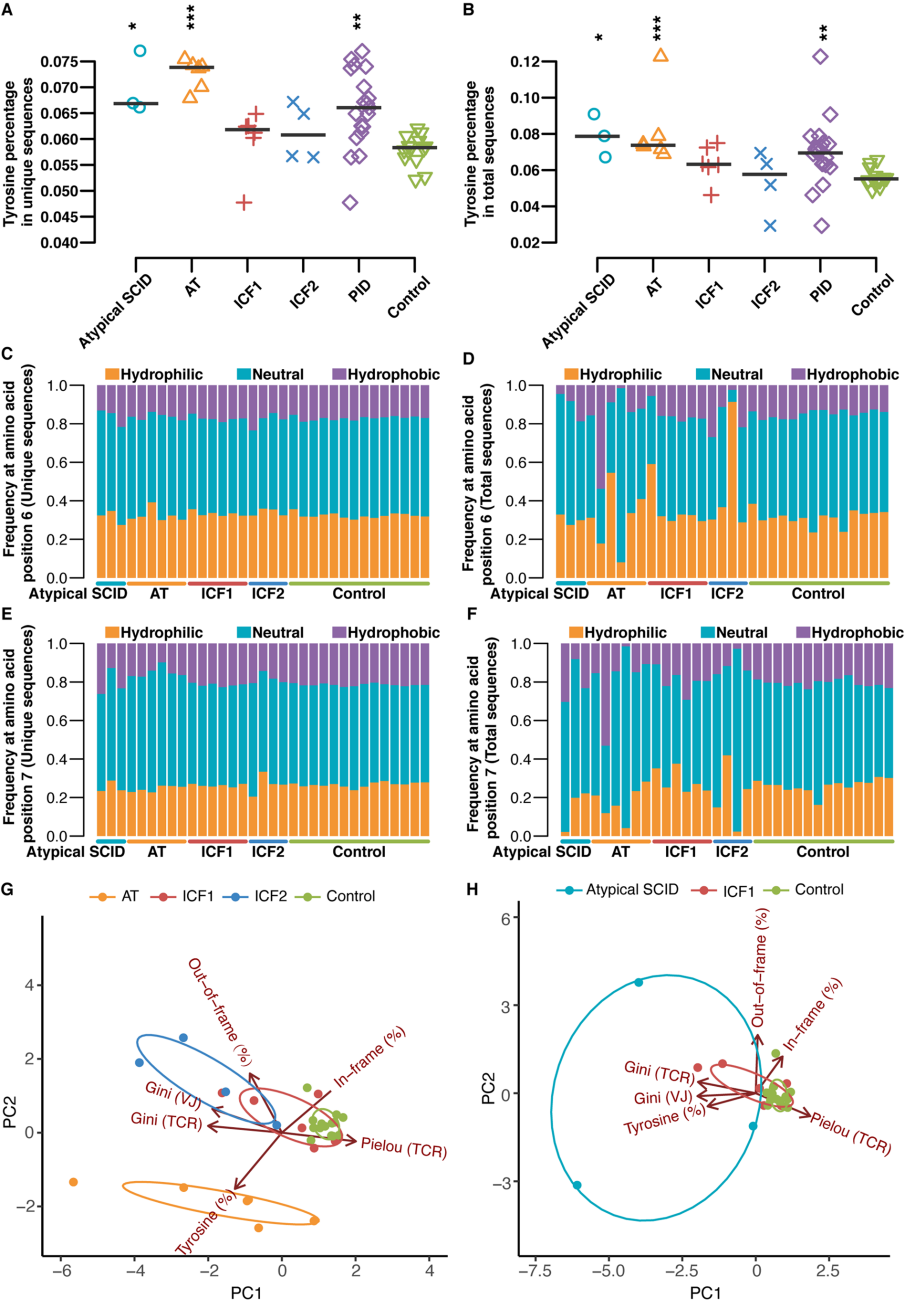
Abnormal amino acid composition of CDR3 in the TCRβ sequences. Percentage of tyrosine residues (Y-Index) in the CDR3 of unique (**A**) and total (**B**) sequences. Amino acid composition of CDR3 in patients and healthy controls for amino acid positions 6 (unique (**C**) and total (**D**)) and 7 (unique (**E**) and total (**F**)) of the 13-amino acid-long CDR-B3. Sample plots illustrating the segregation of the AT and ICF2 patient groups from healthy controls (**G**) whereas ICF1 and atypical SCID patient groups cannot separate from each other nor controls (**H**) based on principal component 1 (PC1) and PC2 based on six variables (Pielou’s evenness (TCR), Gini skewing index (TCR), Gini skewing index (V-J paring), the percentage of in-frame rearrangement, mean CDR3 length of out-of-frame rearrangement, and the percentage of tyrosine in unique clones. The asterisk above each group indicates the significance of tests between each group and normal controls (*p* ≤ 0.05 *, *p* ≤ 0.01 **, *p* ≤ 0.001 ***, *p* ≤ 0.0001 ****, two-sided Wilcoxon Rank Sum Test followed by multiple test correction)

Hydrophobicity of positions 6 and 7 amino acids of the 13-amino acid-long CDR-B3 promotes the development of self-reactive T cells [52], as do the aromatic residues in CDR3 sequences enriched by positively charged tyrosine(s) [53]. Notably, the percentage of tyrosine residues in the CDR3 is significantly higher in AT patients (Fig. 4A–B) and also shows an appreciable divergence of hydrophobic amino acid usage at positions 6 and 7 of total clone sequences in AT and ICF2 patients (Fig. 4C–F), suggesting that the peripheral population in these patients differs from healthy controls.

Moreover, previous studies have shown that TdT preferentially uses dGTP and dCTP as compared to dAMP or dTMP during generation of junctional diversity which results in a high G/C content of the TCR insertion regions [4, 48, 54]. Clear distinctions were observed for the average percentage of inserted nucleotides in the sequence which is consistent with this notion in IEI patients or normal individuals (Table S5). However, no significant differences were observed for the average GC content among the different patient groups and normal individuals (Fig. S7).

### Unsupervised Clustering of TCR Parameters Segregates AT and ICF2 Patients

Principal components analysis (PCA) was performed in order to assess whether the characteristics of the TCRβ repertoire could distinguish patients from normal individuals. The characteristics in patients with ATM deficiency, ICF2 patients, and healthy donors were distinct based on six variables (Pielou’s evenness (TCR), Gini skewing index (TCR), Gini skewing index (V-J paring), the percentage of in-frame rearrangement, mean CDR3 length of out-of-frame rearrangement, and the percentage of tyrosines in unique clones) (Fig. 4G). On the other hand, the ICF1 group and the atypical SCID patients could not be differentiated from each other, and the ICF1 patients clustered together with the ICF2 group and the normal individuals (Fig. 4G–H).

### Discrepant Enrichment of Pathology-Associated T Cells Contributes to the Phenotypic Heterogeneity of Immunodeficiencies

The phenotypic heterogeneity of immunodeficiencies is well established. For instance, opportunistic infections are rarely reported in AT patients [55–57] but have been identified in several ICF patients [12, 58]. Furthermore, AT patients also have an increased risk of developing autoimmune diseases, including immune thrombocytopenia (ITP), several forms of arthritis, and vitiligo [59, 60]. Moreover, AT patients are predisposed to lymphoid malignancies [61]. However, ICF patients rarely develop cancer [62].

To further investigate the association between the TCR repertoire abnormality and the heterogeneous symptoms/complications in AT and ICF patients, we performed a comprehensive analysis of the enrichment of literature which reported pathology-associated TCR (paTCR) clonotypes within each patient group (see “Materials and Methods” section).

**Fig. 5:**
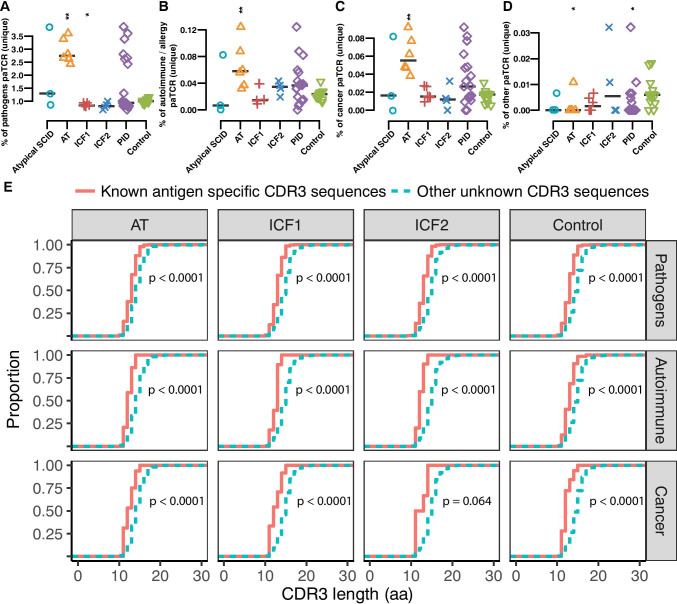
AT patients display a high percentage of pathology associated and common clonotypes. Proportion of literature reported pathology-associated TCR (paTCR) clonotypes in unique CDR3 sequences in each patient group (**A**–**D**). AT patients have higher percentage of pathogens (**A**), autoimmune and allergy (**B**), cancer (**C**) associated and low percentage of other (**D**) reported TCR clones, whereas ICF1 and ICF2 patients have a low percentage of pathogen-associated clonotypes. **E** CDR3 length distribution in known pathology-associated TCR and unknown clonotypes (one-sided bootstrapped Kolmogorov–Smirnov test). The asterisk above each group indicates the significance of tests between each group and normal controls (*p* ≤ 0.05 *, *p* ≤ 0.01 **, *p* ≤ 0.001 ***, *p* ≤ 0.0001 ****, two-sided Wilcoxon Rank Sum Test followed by false discovery rate control)

The proportion of known paTCR CDR3s out of all unique CDR3s in a sample may represent the abundance of paTCRs in the pre-selected repertoire, and the proportion of paTCRs in total CDR3s includes the influence of the selection process. We found that the proportion of known pathogen, autoimmunity, and cancer-associated clonotypes was significantly increased among the unique clones in the AT group (Fig. 5A–D and Fig. S8 A-I). However, there were no significant differences in a fair number of inflammatory diseases associated with clonotypes between AT patients and controls; these diseases include aseptic meningitis, transverse myelitis, polyradiculitis, inflammatory cranial neuropathy, and muscular dystrophy. Besides, only a significant increment of multiple sclerosis (MS)-specific clonotypes (Fig. S8H) out of autoimmune disease in AT patients and melanoma-specific clonotypes out of cancer-associated clonotypes enriched in AT patients (Fig. S8I, L), suggesting that the proportion of pathology-associated clones in the AT patients were not undifferentiated. As for the high proportion of melanoma-specific clonotypes observed in AT group, other studies also showed that mutations in *ATM* were associated with the risk of melanoma [63], and the change in phospho-ATM expression was associated with melanoma progression [64].

We furthermore checked the correlation between the frequency of pathology-associated clonotypes with the TCR diversity Shannon index, but no significant association among them was found (Fig. S9, A-C), implying that changes in the proportion of known paTCR CDR3s were independent of global TCR diversity and may independently reflect an increased risk of AT-associated complications.

In contrast to AT patients, a low frequency of pathogen-specific clonotypes was recorded in the ICF1 patients (Fig. 5A). Interestingly, ICF1 and ICF2 groups both showed a lower proportion of common clonotypes, which were defined as clonotypes presented in 5 or more control samples in our study (Fig. 5A–D and Fig. S8, A-I).

To test the contribution of antigen selection to the over-representation of paTCRs, we also compared the proportion of paTCRs in the total CDR3s among each group (Fig. S8, K-M**)**. The proportion of pathogen and cancer paTCRs in the total CDR3s in the AT patients was also significantly higher than in healthy controls (Fig. S8, K-L). However, less magnitude of enrichment was observed in the total CDR3s compared to the unique hits, for both cancer and autoimmunity associated clones in the AT patients, perhaps reflecting that none of the pediatric AT patients in this study had as yet developed cancer or autoimmune disease. The frequency of pathogen-specific TCRs among the total clones fluctuated widely (Fig. S8M), indicating that the infection status might be different in each individual. Taken together, we propose that the over-representation of paTCRs in the pre-selected TCR repertoire contributes to the discrepant symptoms of AT patients compared to the ICF patients.

We supposed that the change of paTCR proportion in patients may be related to the shortening of CDR3 length. In order to examine this notion, we compared the length distribution of known pathology-specific clonotypes with those unknown clonotypes in each group. The known pathology-associated TCR showed a dramatically higher proportion of short CDR3 length compared to other unknown clonotypes (Fig. 5E). Therefore, the enrichment of paTCR might occur in the process of abnormal VDJ recombination which produces TCR repertoire with reduced CDR3 length.

## Discussion

We performed WES and deep sequencing of the CDR3 regions of TCRβ in 19 IEI patients with DNA repair/methylation defects. Variable TCR repertoire characteristics in different disease groups were revealed based on genomic data comparison with healthy donors. The finding provides clues to address the function of ATM, DNMT3B, and ZBTB24 proteins during V(D)J recombination.

Mutated genes found in atypical SCID patients might lead to different immune repertoire alterations, and different immune repertoire change has been observed in other SCID patients [23]. Reduced clonality in *RAG1* deficient patients (P1) was not always caused by skewed V-J pairing, which is consistent with a report that patients with RAG deficiency present diverse immune repertoire characteristics [23]. Furthermore, our findings did not show that the immune repertoire of patient with the *JAK3* mutation (P3) was markedly altered as compared to controls, which is reasonable since JAK3 is not directly involved in the V(D)J recombination process.

ATM function is important for both the NHEJ and HR pathways of DSB repair [65]. Biallelic mutant ATM mice die during early embryonic life, whereas heterozygous embryonic stem cells show a higher genomic instability and inhibited DSB repair which subsequently blocks lymphocyte development [65]. Our findings confirm and extend previous observations of ATM function in DNA repair pathways during V(D)J recombination [66]. The T cell repertoire in our AT patients was characterized by a markedly reduced diversity, in agreement with previous studies suggesting that decreased thymic output and increased proliferation restrict the TCR repertoire in AT patients [39] and a defective TCRβ rearrangement in *Atm* knockout mice [67]. The decreased percentage of in-frame recombinations observed in our study reinforces previous notions that Atm deficiency results in an increased genomic instability [65], decreased TCR V_α_-J_α_ rearrangement, and a defect in thymocyte maturation [68].

Notably, we have found that the shortening of CDR3 length of the AT patients predominantly happens before positive and negative selection in the thymus and that a short CDR3 length in the productive TCR is subsequently modified by selective forces. This suggests a combined result of TCR rearrangement, thymic selection, and antigen-specific expansion, to the CDR3 shortening. We and others [43] have observed that productive TCRs tend to be of a conserved range of length after selection due to a potential selective MHC-I or MHC-II restricted selection process. As the difference of the enrichment before and after selection was not significant between the different groups, these results should be interpreted with caution since this may be due to the limited sample size of each group in our study.

Consistent observations of low numbers of N-nucleotides in the ends of the junctions in AT patients have also been reported previously both in human umbilical cord blood samples [40], neonatal mice [69, 70] as a result of low TdT activity during the fetal period, and antigen receptor development in TdT deficient mice [71], suggesting that low expression or dysfunction of TdT might be one of the downstream consequences of ATM deficiency. This might also contribute to a higher proportion of out-of-frame TCRs with a frameshift present in the AT patients. The small size of the insertions in AT patients might lead to a shorter CDR3 length and accidentally result in a higher occurrence of shared clonotypes. This event underlies the unexpected bias of shorter CDR3 lengths in AT patients, in contrast to neither significant overall CDR3 length change nor consistent InDel length change between ATM knockout and wildtype mice reported in a prior study [67].

AT patients share enriched identical clonotypes with normal individuals and a high proportion of pathology-associated CDR3 sequences that are complementary to pathogen-specific and cancer-associated epitopes. We reasoned that this phenomenon could be due to a recombination bias that lowers the numbers of insertions, where shorter CDR3 lengths by chance increase the likelihood of shared clonotypes; a similar pattern has been seen both in patients with Omenn syndrome caused by *RAG1/2* mutations [22] and in CVID patients [45]. The finding is consistent with previous reports showing a significant decrease of insertion length in highly shared TCR pools and thus a lower number of insertions providing a higher chance of generation of identical CDR3s [45, 50, 51].

**Fig. 6:**
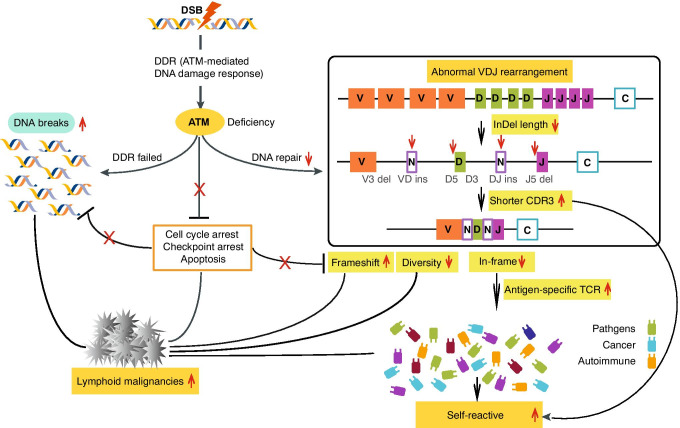
Schematic diagram depicts ATM function in DNA repair pathway and cancer development

To our knowledge, this is the first study to comprehensively and systemically illustrate TCRβ recombination in AT patients. Our results provide a compelling evidence of the role of ATM in tumorigenesis and the development of autoimmune symptoms (Fig. 6). Abnormality of CDR3 amino acid composition and shorter CDR3 length might contribute to the susceptibility of auto-reactivity which has been proposed in type 1 diabetes [43], immunoglobulin A nephropathy [72], and systemic lupus erythematosus [73] and provide an explanation for the autoimmune symptoms observed in AT patients [59, 60, 74]. It is speculated that defective DNA damage repair in AT patients may increase genomic instability, and subsequently, TdT dysfunction which leads to accumulation of out-of-frame rearrangements. ATM defective patients are unable to recognize the key proteins involved in cell cycle arrest and apoptosis [61]. Therefore, AT patients are predisposed to lymphoid malignancies, particularly lymphoma and leukemia [61]. Reduced levels of total T cells and restricted TCR diversity have been previously shown in B cell lymphoma as well [75].

Although recurrent infections in ICF patients are mainly due to B cell defects and impairment of antibody production, opportunistic infections have been reported in several ICF patients, suggesting a subtle T cell dysfunction [12, 58]. However, no significant differences in the immune repertoire except an increase in CDR3 length were observed in the ICF1 patients as compared to normal individuals. Diverse V gene usage and V-J pairing suggest that V(D)J rearrangements proceed normally in ICF1 patients. These results are consistent with the normal T lymphocyte subpopulations observed in our ICF1 subjects (Table 1 and Fig. S1) and in other ICF1 patients where T cell defects have rarely been reported [58]. A similar observation was previously reported for TCRβ5 rearrangements in *Dnmt3a* knockout mice [76] and B cell differentiation in ICF1 patients [77]. One possible interpretation for the low percentage of out-of-frame rearrangements that contain stop codons observed in the ICF1 patients is that it might be a consequence of the nonsense-mediated decay suppression which is regulated by *DNMT3B* [40].

Preferential enrichment of longer CDR3s in ICF patients potentially decreases the number of antigen-specific clonotypes and results in an increased risk for opportunistic infections. Unlike patients with AT, ICF patients rarely develop cancer [62]. ICF syndrome T cells are more likely to undergo spontaneous apoptosis, which possibly prevents tumor cell development from cytogenetically abnormal ICF lymphocytes [78]. Our findings on the T cell repertoire features in ICF1 patients support the previous hypothesis that hypomethylation indirectly affects T-lymphocyte function [77] and subsequently the clinical features.

ZBTB24 is an essential transcription factor involved in the proliferation and differentiation of B cells by regulating the cell cycle progression [79]. The longer CDR3 lengths result in a low frequency of common clonotypes in the ICF2 patients as compared to public immune repertoire databases. The limited repertoire may reflect altered T cell signaling which in turn promotes immune dysregulation. The current mainstream hypothesis proposes that the ZBTB24 protein, via regulation of DNMT3B expression, could play a role in DNA methylation, gene expression, and regulation of chromatin structure in lymphocyte populations that are important for generation of the immune repertoire in lymphocytes. The significant difference in TCR diversity of patients with ICF1 and ICF2 suggests that ZBTB24 and DNMT3B might bind to different partners in the regulation of DNA methylation and lymphocyte epigenetic modification, contrary to the previous speculation of ZBTB24 and DNMT3B forming a complex during regulation of DNA methylation. It is consistent with the finding that ZBTB24 modulates methylation via CDCA7 in murine cells [80].

Observed skewed clonal proliferation in AT (*TRBV19*) and ICF2 (*TRBV7–*3) also need to be considered as an important immunological “signature.” Although skewed TCR repertoire can result from restricted proliferation, however, specific alterations have been reported in certain infections, malignancies, or immunological disorders. The absence of certain clonal usage in the context of immunodeficiency may hint toward susceptibility to a range of pathogens [81].

Our work suggests the aberrations of TCRβ rearrangement in DNA repair/methylation deficient patients with distinct repertoire characteristics, which offers insight into the pathological mechanisms of different DNA repair/methylation defects during V(D)J recombination and their heterogeneous clinical manifestations. However, it should be noted that the aberrations in repertoire diversity in this study are inferred with a case–control design with a limited sample size; an independent case–control cohort design can presumably generate different overall patterns in terms of some repertoire metrics. This is even more challenging considering other sources of variability in clonotype abundance from repertoire sequencing experiments (biological repertoire variations and fictitious deviation due to noise).

## Supplementary Information

Below is the link to the electronic supplementary material.

Supplementary file1 (DOCX 6611 kb)

## Data Availability

The data for this study have been deposited in the database of CNGB Nucleotide Sequence Archive (CNSA, https://db.cngb.org/cnsa/) with accession no. CNP0000138.

## Code Availability

IMonitor: https://github.com/zhangwei2015/IMonitor
