# Supplementary material for: T Cell Repertoire Abnormality in Immunodeficiency Patients with DNA Repair and Methylation Defects
Source: J Clin Immunol. 2021 Nov 25;42(2):375–93. doi: 10.1007/s10875-021-01178-1 (PMC8821531; doi:10.1007/s10875-021-01178-1)
Supplement: Supplementary file 1 — Supplementary file1 (DOCX 6611 kb) [file 10875_2021_1178_MOESM1_ESM.docx]

**SUPPLEMENTARY MATERIAL**

**T cell repertoire abnormality in immunodeficiency patients with DNA repair and methylation defects**

Fang *et al*.

**
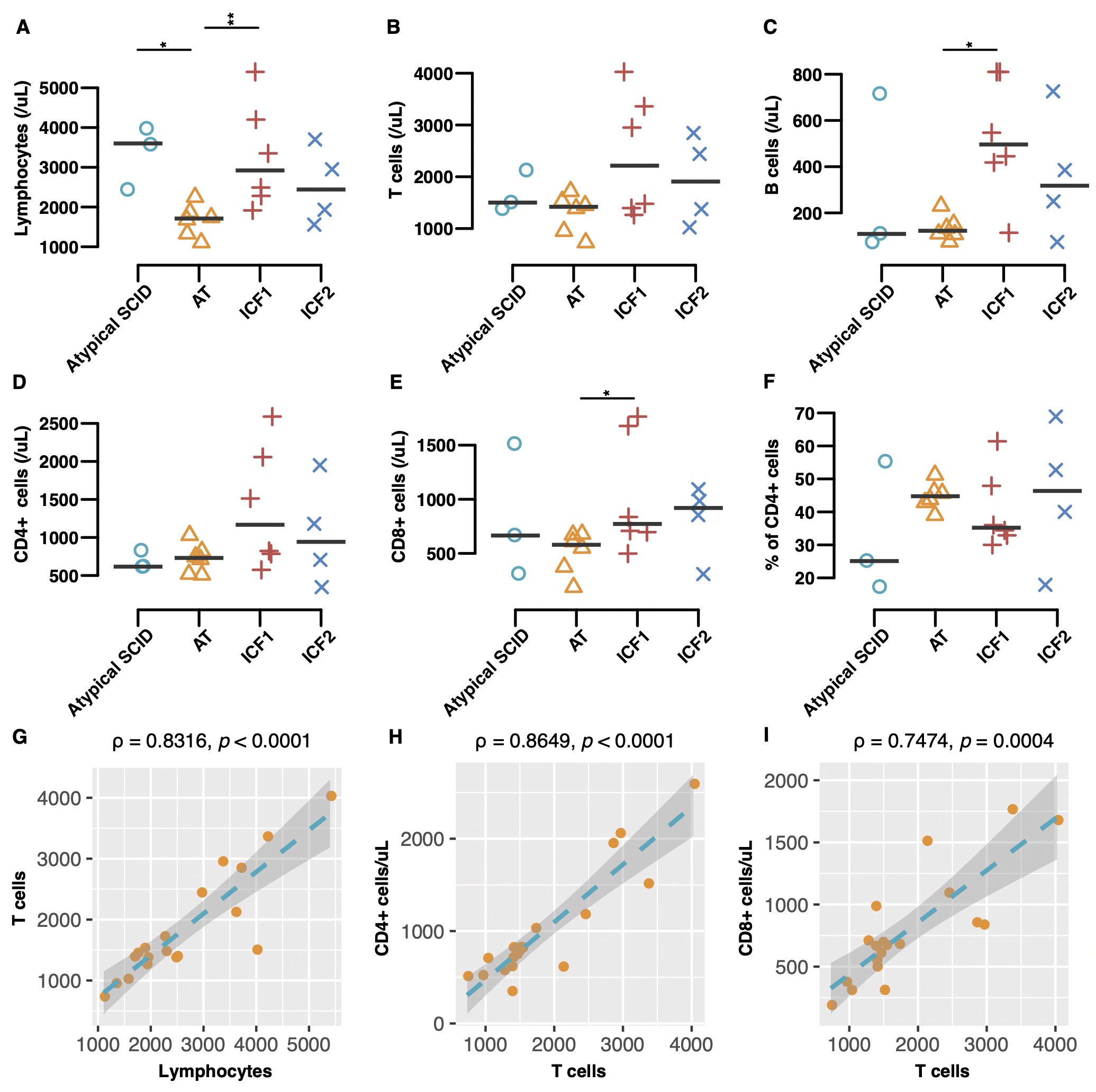
**

**Figure S1. Immunologic profile of studied subjects and correlation analysis.** **A-F** Peripheral blood subpopulations were enumerated in a scatter plot. **G-I** Correlations between lymphocytes subsets (Spearman tests). Asterisk above each group indicate the significance of tests between each group and normal controls (*p* ≤ 0.05 *, *p* ≤ 0.01 **, *p* ≤ 0.001 ***, *p* ≤ 0.0001 ****, two-sided Wilcoxon Rank Sum Test).

**Figure S2. Skewed usage of V genes in the TCRβ repertoires of patients with DNA repair/methylation defects.** The box plot shows the significant differential usage of V gene in TCRβ repertoires of patients with AT **(A)** and ICF2 **(B)** compared to controls. The asterisk above each group indicates significance tests between each group and normal controls (*p* ≤ 0.001 ***, Bonferroni correction was used for multiple tests correction).

**Figure S3. Tree maps show the diversity of TCRβ repertoires from patient groups and normal individuals.** Each rectangle indicates a specific V-J clone, and the size of the rectangle corresponds to its frequency, random colors were filled in rectangles to make them distinctive.

**Figure S4. Altered CDR3 length in patients compared to healthy controls.**

**A** In-frame TCRβ sequences in ICF1 have longer CDR3s than normal controls and shorter CDR3s in atypical SCID compared to healthy controls. **B** Longer pre-selection CDR3 sequences in ICF1 and ICF2 patients compared to healthy individuals. **C, D, E** Mean length of V, D, J gene, mean length of V gene of CDR3 region is significantly raised in AT, ICF1 and ICF2 patients (**C**), whereas only AT patients reached statistical significant for D gene length. **F**. Short CDR3s are enriched during thymic selection in all groups (paired t-test). The asterisk above each group indicate the significance tests between each group and normal controls (*p* ≤ 0.05 *, *p* ≤ 0.01 **, *p* ≤ 0.001 ***, *p* ≤ 0.0001 ****, two-sided Wilcoxon Rank Sum Test followed by multiple test correction).

**Figure S5. AT patients present altered InDel distribution during TCRβ rearrangement (Out-of-frame).** Unique out-of-frame sequences of pre-selection clonotypes were analyzed for the distribution of deletions and insertions at each of the 6 rearrangement sites. 3’V (**A**), 5’D (**B**), 3’D (**C**) and 5’J (**D**) deletion, V-D (**E**) and D-J (**F**) insertion. One-sided Wilcoxon Rank Sum Test revealed that AT patients were prone to have short deletions (**A-D**) and even shorter insertions (**E, F**).

**Figure S6. AT patients present altered InDel distribution during TCRβ rearrangement (In-frame).** Unique in-frame sequences of post-selection clonotypes were analysed for the distribution of deletions and insertions at each of the 6 rearrangement sites. 3’V (**A**), 5’D (**B**), 3’D (**C**) and 5’J (**D**) deletion, V-D (**E**) and D-J (**F**) insertion. One-sided Wilcoxon Rank Sum Test revealed that AT patients are prone to have short deletions (**A-D**) and even shorter insertion (**E, F**). One-sided Wilcoxon Rank Sum Test for the significance of length difference between each group pair.

**Figure S7. Distribution of nucleotides in the insertion region in DNA repair/methylation defect patients and controls.** The box plots depict the frequency of insertion of Adenosine **(A)**, Thymidine **(B)**, Cytosine **(C)**, Guanine **(D)** and GC total insertions **(E)** (two-sided Wilcoxon Rank Sum Test).

**
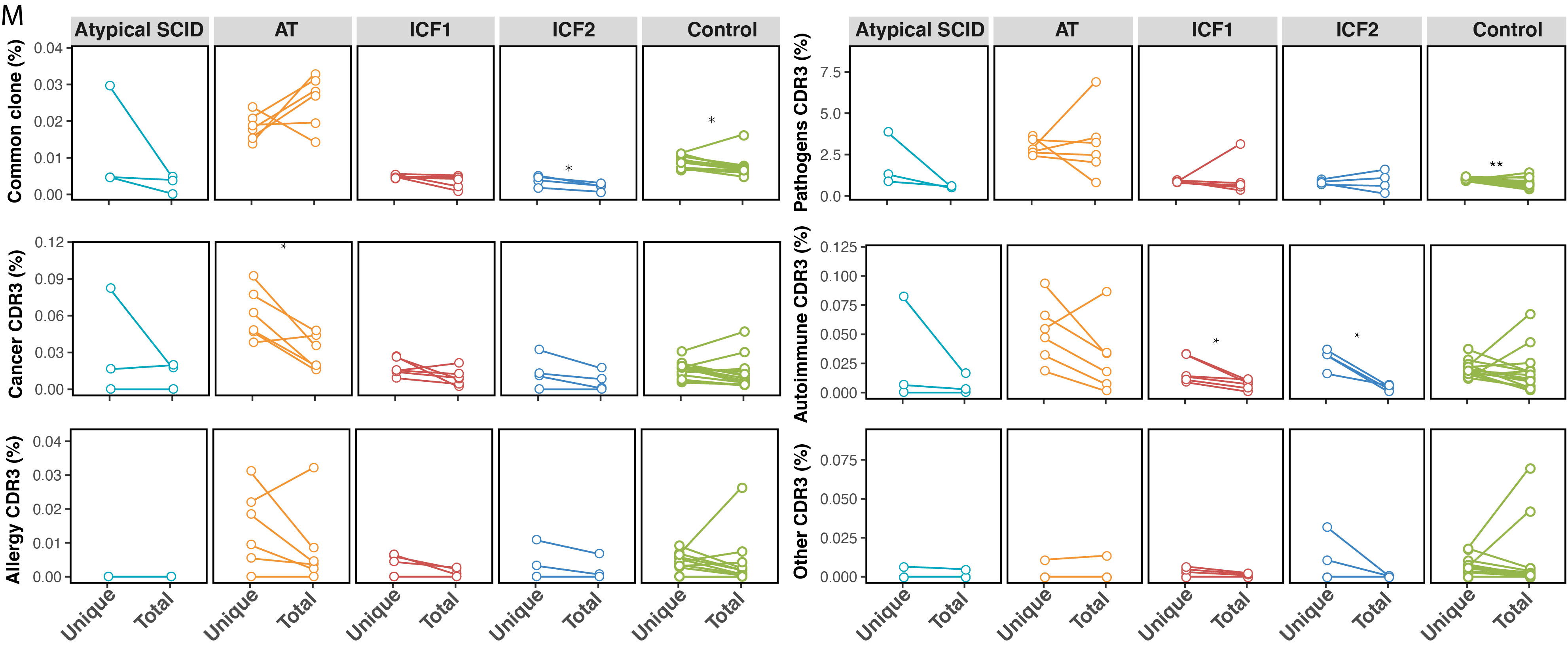
**

**Figure S8. AT patients have a high percentage of pathogens, autoimmune disease and cancer associated clones.** Proportion of clones associated with different pathogens, including influenza **(A),** CMV **(B)**, DENV **(C)**, yellow fever **(D)**, EBV **(E)**, HIV **(F)**, tuberculosis **(G)**, MS **(H)** and melanoma (**I**) were visualized. (**J**-**L)** presented the percentage of common clones, which are defined as clones present in 5 or more control samples, pathogen and cancer associated clones in total CDR3 sequences of different groups. (**M)** line plots show the percentage of pathology associated clones and common clones in unique and total CDR3 sequences in each patient.

CMV: Cytomegalovirus; DENV: Dengue virus; HIV: Human immunodeficiency virus (HIV) related; EBV: Epstein Barr Virus; MS: Multiple Sclerosis. The asterisk above each group indicates the significance tests between each group and normal controls (*p* ≤ 0.05 *, *p* ≤ 0.01 **, *p* ≤ 0.001 ***, *p* ≤ 0.0001 ****, two-sided Wilcoxon Rank Sum Test followed by multiple test correction).

**
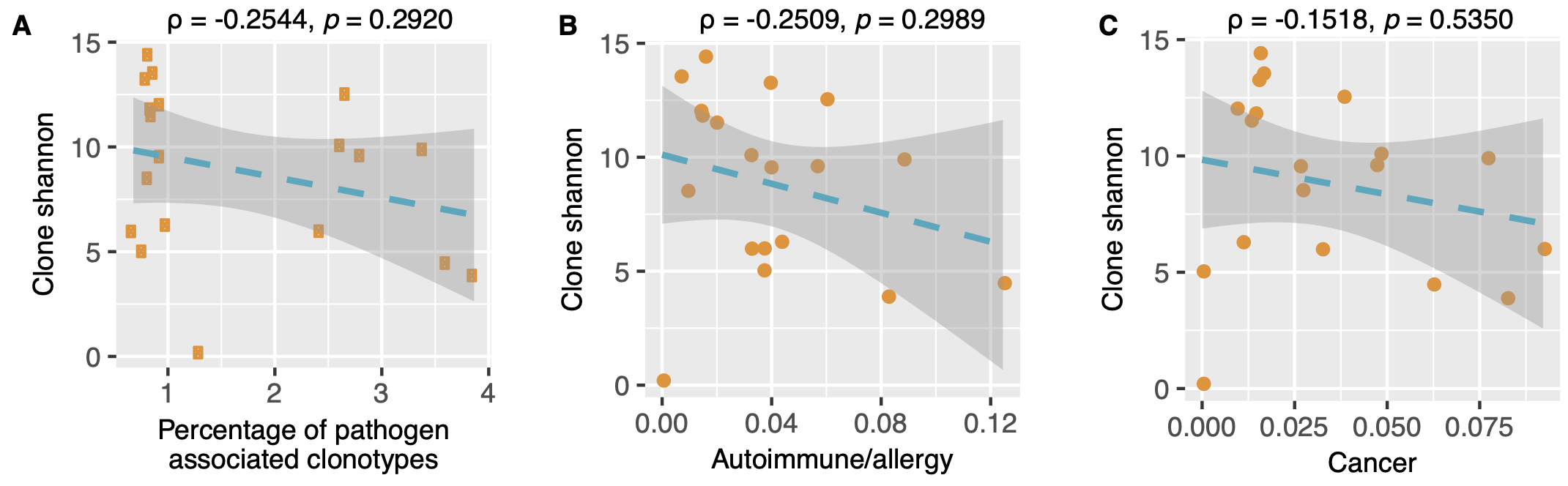
**

**Figure S9. Associations between repertoire diversity and pathology associated clonotypes percentage (Spearman tests)**.

**Table S1. Summary of functional clonotypes.**

| **Category** | **Record No.** | | **Disease Name** |
| --- | --- | --- | --- |
| Pathogens | | 5975 | Influenza infection |
|  |  | 5722 | Cytomegalovirus (CMV) infection |
|  |  | 2481 | Human Immunodeficiency Virus (HIV) infection |
|  |  | 2372 | Epstein Barr Virus (EBV) infection |
|  |  | 1834 | Tuberculosis |
|  |  | 465 | Yellow Fever Virus infection |
|  |  | 454 | Hepatitis C Virus (HCV) infection |
|  |  | 275 | Dengue Virus 1 (DENV) infection |
|  |  | 62 | Tropical Spastic Paraparesis (HTLV-1 retrovirus) infection |
|  |  | 30 | Herpes Simplex Virus 1 (HSV1) infection |
|  |  | 29 | Tick-Borne Encephalitis (TBE) infection |
|  |  | 2 | Reversal Leprosy (*Mycobacterium leprae*) infection |
| Autoimmune | | 213 | Multiple Sclerosis (MS) |
|  |  | 199 | Systemic Lupus Erythematosus (SLE) |
|  |  | 44 | Type 1 Diabetes (T1D) |
|  |  | 31 | Cutaneous Sarcoidosis |
|  |  | 10 | Celiac Disease |
|  |  | 9 | Autoimmune Hepatitis (AIH) |
|  |  | 3 | Churg Strauss Syndrome |
|  |  | 3 | Myasthenia Gravis (MG) |
| Cancer | | 406 | Melanoma |
|  |  | 27 | Lung Cancer |
|  |  | 17 | Breast Cancer |
|  |  | 6 | Leukemia |
|  |  | 3 | Carcinoma |
|  |  | 2 | Hepatocellular Carcinoma |
|  |  | 1 | Carcinoma of Uterine Tube |
|  |  | 1 | Malignant Neoplasm of Pancreas |
|  |  | 1 | Ovarian Cancer |
| Allergy | | 58 | Allergy |
| Other | | 31 | Aseptic Meningitis |
|  |  | 17 | Transverse Myelitis |
|  |  | 13 | Polyradiculitis |
|  |  | 11 | Inflammatory Cranial Neuropathy |
|  |  | 7 | Duehenne’s Muscular Dystrophy |
| **Total** | | **20814** |  |

**Table S2. Metrics summary of TCR diversity in all subjects.**

| **Sample_ID** | **Group** | **Unique Clone** | **Shannon's H (TCR)** | **Pielou's eveness (TCR)** | **Gini skewing index (TCR)** | **Clone_CR4** | **Top100 Clone** | **High Clone Number** | **Gini skewing index (VJ)** | **Pielou's eveness (VJ)** | **VJ_CR4** | **Frequency of in-frame (%)** | **Frequency of out-of-frame (stop_codon) (%)** | **Frequency of out-of-frame (CDR3_length) (%)** | **Frequency of non-function (%)** | **Average of GC percentage (%)** |
| --- | --- | --- | --- | --- | --- | --- | --- | --- | --- | --- | --- | --- | --- | --- | --- | --- |
| P1 | Atypical SCID (RAG1) | 843 | 0.3304 | 0.0340 | 0.9208 | 1.121 | 3.016 | 8 | 0.8694 | 0.0408 | 1.0500 | 85.81 | 1.65 | 12.44 | 0.1 | 0.5827 |
| P2 | Atypical SCID (DCLRE1C) | 3599 | 3.8534 | 0.3262 | 0.9444 | 76.729 | 88.207 | 33 | 0.9469 | 0.3346 | 80.5244 | 58.32 | 1.33 | 38.13 | 2.22 | 0.6866 |
| P3 | Atypical SCID  (JAK3) | 30059 | 13.5014 | 0.9076 | 0.6600 | 0.873 | 7.723 | 10 | 0.7857 | 0.7632 | 25.5236 | 81.49 | 3.42 | 14.91 | 0.18 | 0.5933 |
| P4 | AT | 3178 | 4.4542 | 0.3829 | 0.9258 | 66.146 | 83.504 | 50 | 0.9239 | 0.3989 | 69.5780 | 38.82 | 1.07 | 58.37 | 1.74 | 0.5261 |
| P5 | AT | 18059 | 12.5046 | 0.8843 | 0.6917 | 2.324 | 16.558 | 72 | 0.7116 | 0.8435 | 10.2206 | 69.35 | 3.22 | 25.2 | 2.24 | 0.5850 |
| P6 | AT | 10481 | 9.5760 | 0.7170 | 0.8283 | 18.121 | 51.629 | 85 | 0.7955 | 0.7652 | 21.1144 | 73.63 | 2.29 | 21.95 | 2.13 | 0.5833 |
| P7 | AT | 12276 | 10.0637 | 0.7409 | 0.8126 | 19.707 | 44.973 | 93 | 0.7748 | 0.7841 | 19.5642 | 74.41 | 3.5 | 21.5 | 0.59 | 0.5791 |
| P8 | AT | 9030 | 9.8686 | 0.7510 | 0.7755 | 21.174 | 42.687 | 90 | 0.7446 | 0.7699 | 24.8904 | 67.51 | 3.93 | 28.09 | 0.47 | 0.5734 |
| P9 | AT | 5416 | 8.5849 | 0.6922 | 0.8518 | 26.229 | 57.300 | 120 | 0.7973 | 0.7270 | 32.2014 | 64.55 | 9.28 | 25.63 | 0.54 | 0.6143 |
| P10 | ICF1 | 10881 | 8.4756 | 0.6321 | 0.8643 | 26.290 | 65.319 | 68 | 0.8580 | 0.6946 | 26.6802 | 81.37 | 3.07 | 15.33 | 0.23 | 0.5933 |
| P11 | ICF1 | 21449 | 11.9983 | 0.8339 | 0.7145 | 9.754 | 28.019 | 64 | 0.7404 | 0.8238 | 12.5158 | 78.84 | 3.1 | 17.42 | 0.64 | 0.5895 |
| P12 | ICF1 | 32795 | 13.2228 | 0.8814 | 0.6441 | 6.585 | 17.436 | 27 | 0.7503 | 0.8255 | 9.5218 | 78.74 | 2.66 | 18.03 | 0.57 | 0.5850 |
| P13 | ICF1 | 14985 | 9.5335 | 0.6873 | 0.7877 | 35.219 | 46.641 | 29 | 0.8235 | 0.6975 | 36.4856 | 85.62 | 3.4 | 10.78 | 0.2 | 0.5818 |
| P14 | ICF1 | 20819 | 11.7877 | 0.8217 | 0.7128 | 10.204 | 30.982 | 58 | 0.7072 | 0.8426 | 11.6032 | 69.97 | 3.07 | 20.12 | 6.84 | 0.5681 |
| P15 | ICF1 | 44193 | 14.3629 | 0.9308 | 0.5936 | 0.301 | 4.452 | 0 | 0.7542 | 0.8038 | 16.9788 | 81.32 | 3.4 | 14.9 | 0.38 | 0.5770 |
| P16 | ICF2 | 8873 | 6.2654 | 0.4777 | 0.9144 | 56.605 | 76.703 | 61 | 0.8882 | 0.6221 | 40.8752 | 71.22 | 15.87 | 12.45 | 0.46 | 0.5728 |
| P17 | ICF2 | 3071 | 7.2296 | 0.6241 | 0.8977 | 34.362 | 71.797 | 114 | 0.8128 | 0.7373 | 25.2352 | 81.55 | 4.22 | 13.96 | 0.27 | 0.6266 |
| P18 | ICF2 | 2680 | 5.0088 | 0.4398 | 0.9304 | 58.977 | 83.556 | 66 | 0.9021 | 0.4797 | 60.0608 | 88.41 | 1.55 | 9.87 | 0.17 | 0.6272 |
| P19 | ICF2 | 29860 | 11.4618 | 0.7710 | 0.7370 | 15.546 | 38.952 | 62 | 0.7644 | 0.7960 | 18.9886 | 79.99 | 3.24 | 14.55 | 2.21 | 0.5929 |
| C1 | Control/  Control-PM | 28450 | 13.4611 | 0.9098 | 0.6405 | 2.493 | 10.340 | 24 | 0.7553 | 0.8062 | 16.4138 | 86.4 | 2.78 | 10.52 | 0.3 | 0.5966 |
| C2 | Control/  Control-PM | 35805 | 14.0679 | 0.9299 | 0.5982 | 0.421 | 5.985 | 3 | 0.6886 | 0.8596 | 8.0404 | 79.62 | 3.39 | 16.44 | 0.55 | 0.5887 |
| C3 | Control/  Control-PM | 36230 | 13.7090 | 0.9052 | 0.6361 | 3.624 | 9.666 | 12 | 0.7806 | 0.7826 | 20.0634 | 84.24 | 3.35 | 11.97 | 0.44 | 0.5803 |
| C4 | Control | 16849 | 12.6425 | 0.9004 | 0.6619 | 1.899 | 13.171 | 65 | 0.7295 | 0.8074 | 17.2670 | 80.72 | 4.62 | 14.03 | 0.62 | 0.5884 |
| C5 | Control | 16379 | 12.3724 | 0.8838 | 0.6873 | 3.508 | 17.039 | 65 | 0.7605 | 0.7964 | 15.6862 | 81.88 | 4.36 | 13.28 | 0.47 | 0.5718 |
| C6 | Control | 31268 | 13.4742 | 0.9023 | 0.6010 | 6.642 | 12.108 | 11 | 0.7121 | 0.8272 | 15.2872 | 76.6 | 3.92 | 18.75 | 0.73 | 0.5935 |
| C7 | Control | 15542 | 12.5765 | 0.9032 | 0.6570 | 1.101 | 12.613 | 78 | 0.7497 | 0.8001 | 17.1064 | 77.28 | 4.58 | 17.73 | 0.41 | 0.6121 |
| C8 | Control | 12622 | 12.1596 | 0.8925 | 0.6795 | 1.840 | 15.921 | 113 | 0.7461 | 0.7975 | 16.8170 | 81.22 | 4.23 | 12.71 | 1.84 | 0.5681 |
| C9 | Control | 16076 | 12.0276 | 0.8608 | 0.7033 | 8.233 | 20.701 | 57 | 0.7676 | 0.7895 | 19.8012 | 82.14 | 3.41 | 13.86 | 0.58 | 0.5441 |
| C10 | Control | 27730 | 13.5853 | 0.9205 | 0.6235 | 1.000 | 7.344 | 7 | 0.7824 | 0.7790 | 18.1916 | 82.91 | 4.18 | 12.73 | 0.18 | 0.5806 |
| C11 | Control | 16857 | 12.2033 | 0.8691 | 0.6669 | 8.481 | 21.816 | 47 | 0.7279 | 0.8194 | 14.2334 | 75.86 | 4.38 | 17.1 | 2.67 | 0.5961 |
| C12 | Control | 18561 | 12.0735 | 0.8514 | 0.6624 | 13.442 | 21.118 | 21 | 0.7502 | 0.7920 | 20.0642 | 81.63 | 4.36 | 13.61 | 0.39 | 0.5788 |
| C13 | Control | 22295 | 13.2025 | 0.9140 | 0.6359 | 1.588 | 9.229 | 21 | 0.7447 | 0.8041 | 17.1388 | 81.83 | 3.95 | 13.71 | 0.51 | 0.5845 |
| C14 | Control | 10775 | 11.2039 | 0.8364 | 0.7079 | 12.872 | 28.674 | 81 | 0.7506 | 0.7923 | 19.7004 | 77.64 | 3.82 | 17.12 | 1.42 | 0.5812 |

**Table S3. Summary of InDel distribution and length difference between patients and controls.**

| **Comparisons** | | **In-frame/out frame InDel** | | ***p* value (One-sided Wilcoxon Rank Sum Test)** | | | | | | | | | | | |
| --- | --- | --- | --- | --- | --- | --- | --- | --- | --- | --- | --- | --- | --- | --- | --- |
|  |  |  |  | **V3** | | **D5** | | **D3** | **J5** | | **V-D** | | **D-J** | | |
| Atypical SCID vs Control | In-frame | | 0.102 (less) | | 0.135 (greater) | | 0.014 (less) | | | < 0.0001 (greater) | | 0.161 (less) | | 0.173 (less) |  |
|  | out-of-frame | | 0.193 (greater) | | 0.259 (greater) | | 0.19 (less) | | | 0.18 (greater) | | 0.154 (less) | | 0.157 (less) |  |
| AT vs Control | In-frame | | 0.009 (less) | | < 0.0001 (less) | | 0.024 (less) | | | < 0.0001 (less) | | < 0.0001 (less) | | < 0.0001 (less) |  |
|  | out-of-frame | | < 0.0001 (less) | | < 0.0001 (less) | | 0.006 (less) | | | < 0.0001 (less) | | 0.001 (less) | | < 0.0001 (less) |  |
| ICF1 vs Control | In-frame | | 0.051 (greater) | | 0.343 (less) | | < 0.0001 (greater) | | | 0.198 (less) | | 0.019 (greater) | | 0.071 (greater) |  |
|  | out-of-frame | | 0.12 (greater) | | 0.192 (less) | | 0.015 (greater) | | | 0.058 (greater) | | 0.025 (greater) | | 0.022 (greater) |  |
| ICF2 vs Control | In-frame | | 0.041 (greater) | | 0.105 (greater) | | 0.113 (less) | | | 0.179 (greater) | | 0.097 (less) | | 0.241 (less) |  |
|  | out-of-frame | | 0.133 (less) | | < 0.0001 (less) | | 0.001 (greater) | | | 0.033 (less) | | 0.215 (greater) | | 0.081 (greater) |  |
| IEI vs Control | In-frame | | 0.339 (less) | | 0.337 (less) | | 0.02 (less) | | | 0.423 (less) | | 0.015 (less) | | 0.023 (less) |  |
|  | out-of-frame | | 0.195 (less) | | 0.017 (less) | | 0.445 (less) | | | 0.057 (less) | | 0.054 (less) | | 0.016 (less) |  |

**Table S4. Decreases in the number of added nucleotides in AT patients.**

| **Group** | **Out-of-frame (insertion length (nt))** | | **In-frame (insertion length (nt))** | |
| --- | --- | --- | --- | --- |
|  | avg_VD | avg_DJ | avg_VD | avg_DJ |
| AT | 2.50 | 2.18 | 1.83 | 1.72 |
| Control | 6.07 | 5.87 | 4.24 | 4.41 |
| p-value* | 0.0002 | 5.16E-05 | 5.16E-05 | 5.16E-05 |

**: Two-sided Wilcoxon Rank Sum Test.*

**Table S5. The average percentage of nucleotide composition for inserted regions in the CDR3.**

| **Group** | **Avg_A_percentage** | **Avg_T_percentage** | **Avg_C_percentage** | **Avg_G_percentage** | **Avg_GC_percentage** |
| --- | --- | --- | --- | --- | --- |
| Control | 22.06% | 19.62% | 28.42% | 29.90% | 58.32% |
| All IEI Patients | 21.59% | 19.26% | 29.44% | 29.71% | 59.15% |
